# Left atrial appendage morphology, echocardiographic characterization, procedural data and in-hospital outcome of patients receiving left atrial appendage occlusion device implantation: a prospective observational study

**DOI:** 10.1186/s12872-016-0200-z

**Published:** 2016-01-28

**Authors:** Christian Fastner, Michael Behnes, Benjamin Sartorius, Mustafa Yildiz, Kambis Mashayekhi, Ibrahim El-Battrawy, Ralf Lehmann, Stefan Baumann, Tobias Becher, Martin Borggrefe, Ibrahim Akin

**Affiliations:** First Department of Medicine, University Medical Center Mannheim, University of Heidelberg, DZHK (German Center for Cardiovascular Research), Mannheim, Germany; Department of Cardiology, Istanbul University Cardiology Institute, Istanbul, Turkey; Internal Medicine Clinic II, Helios Vogtland Klinikum Plauen, Academic Teaching Hospital of Leipzig University, Leipzig, Germany

**Keywords:** Left atrial appendage occlusion device implantation, Echocardiography, Procedural data, In-hospital outcome

## Abstract

**Background:**

Implantation of left atrial appendage (LAA) occlusion devices was shown to be a feasible and effective alternative to oral anticoagulation in patients with non-valvular atrial fibrillation. However, only few data about in-hospital and peri-procedural data are currently available. This study aims to report about echocardiographic, procedural and in-hospital data of patients receiving LAA occlusion devices.

**Methods:**

This single-center, prospective and observational study includes consecutively patients being eligible for percutaneous implantation of LAA occlusion devices (either Watchman™ or Amplatzer™ Cardiac Plug 2). Data on pre- and peri-procedural transesophageal echocardiography (TEE), implantation and procedure related in-hospital complications were collected. The primary efficacy outcome measure was a successful device implantation without relevant peri-device leaks (i.e., < 5 mm).

**Results:**

In total, 37 patients were included, 22 receiving the Watchman™ and 15 ACP 2 device. Baseline characteristics did not differ significantly in both patient groups. The primary efficacy outcome measure was reached in 91.9 % of patients (90.9 % for the Watchman™, 93.3 % for the ACP 2 group). One device embolization (Watchman™ group) with successful retrieval occurred (2.7 % of patients). No thromboembolism or device thrombosis were present. The majority of bleedings was caused by access site bleedings (88.3 % of all bleedings), consisting mostly of mild hematomas corresponding to a BARC type 1 bleeding (80.0 % of all access-site complications). One patient died due to septic shock (non-procedure related).

**Conclusions:**

In daily real-life practice, percutaneous treatment with LAA occlusion devices appears to be an effective and safe.

## Background

Atrial fibrillation (AF) represents the most common cardiac arrhythmia with a current age-dependent prevalence of 1–2 % in the Western population, while being assumed to rise significantly within the next couple of years [1]. Cerebral ischemic stroke represents the most important fatal complication of AF deteriorating the prognosis of each individual patient. In the presence of AF, the risk of stroke increases about 5-times [2], being accompanied by a stroke-related mortality rate of about 20 % [3, 4] and a high percentage of patients remaining disabled. The so called CHA_2_DS_2_-VASc score assesses the patient’s individual annual stroke risk and allows a specific risk-adapted anticoagulant treatment [5].

Oral anticoagulation (OAC) with vitamin K antagonists or with new direct oral anticoagulants (DOACs) prevents effectively thromboembolic events in patients with at least one risk factor [6]. In contrast, major bleedings-particularly cerebral bleedings-still represent a most crucial complication of OAC, despite the fact that DOACs were shown to reduce the occurrence of intracranial hemorrhage [7]. The individual bleeding risk as assessed by the HAS-BLED score [5], contraindications against OAC and the patient’s preference to deny OAC, need to be considered before initiating OAC. In daily clinical practice, half of patients with an increased risk for thromboembolic complications and without any contraindication against OAC does not receive this treatment [8].

The percutaneous implantation of left atrial appendage (LAA) occlusion devices such as the Watchman™ device (Boston Scientific, Natick, MA) and the Amplatzer™ Cardiac Plug (ACP) replaced by the Amplatzer™ Amulet™ (also known as ACP 2, both St. Jude Medical, St. Paul, MN) was evaluated as an effective alternative to OAC in patients with non-valvular AF and concomitant high bleeding risk or in patients being unwilling to take lifelong OAC. The non-inferiority to warfarin therapy in patients being eligible for OAC was proven in the PROTECT-AF trial for the Watchman™ device [9]. Here, warfarin was prescribed for at least 45 days after successful device implantation. In subsequent studies dual antiplatelet therapy (DAPT) for at least 6 months after implantation was shown to be safe in patients with contraindications for OAC [10]. With regard to the ACP device, a non-inferiority study has not yet been investigated. However, DAPT was shown to be safe and effective for this device [11]. First clinical experience with the ACP 2 did not show significant differences compared to the original ACP with regard to peri- and post-procedural complications [12].

Although the implantation of LAA occlusion devices is well established, there is still a great lack of in-hospital and peri-procedural data addressing the safety and efficacy of these devices [12–19]. Specifically, peri-procedural data have been shown to vary tremendously between individual centers. Today, an increase of and peri-procedural safety has developed compared to early implantation periods [13, 14, 20]. Therefore, this study aims to comparatively evaluate in-hospital single-center registry data of patients scheduled for LAA occlusion device implantation (i.e., Watchman™ versus ACP 2).

## Methods

### Enrollment

The present study is a single-center, prospective, observational, descriptional and non-randomized registry including consecutively 37 patients with all three types of non-valvular AF, a CHA_2_DS_2_-VASc score ≥ 2 and, therefore, indication for OAC. Patient enrollment started in June 2014. Further inclusion criteria were age ≥ 18 years, any relative or absolute contraindication for standard OAC - i.e., major bleeding with tendency to recidivity, HAS-BLED score ≥ 3 and neurological symptoms during treatment with OAC or intolerance to OAC. Written informed consent was obtained from all study patients. Patients were excluded if at least one of the following criteria were evident: single episode of AF or treatable reason for AF, catheter ablation of AF within 30 days prior to or after potential LAA occluder implantation, electrical cardioversion within 30 days after potential occluder implantation, congestive heart failure corresponding to functional class NYHA IV, myocardial infarction within the last 3 months, atrial septum defect or interventional/surgical occlusion of ASD, mechanical heart valve, status after heart transplant, symptomatic carotid artery stenosis, transient ischemic attack (TIA) or stroke within last 30 days, intracerebral bleeding within the last 3 months, acute infection, existing or planned pregnancy, existing thrombus. The study was carried out according to the principles of the declaration of Helsinki and was approved by the medical ethics committee II of the Faculty of Medicine Mannheim, University of Heidelberg, Germany.

### Procedure

An electrocardiogram (ECG), standard blood analyses and transesophageal echocardiography (TEE) were performed for pre-procedural planning. LAA occlusion device implantation was performed by experienced interventional cardiologists (≥50 LAA closure device implantations each prior to the study). Patients were treated with conscious sedation using intravenous 2,6-di(propan-2-yl)phenol and midazolam. One arterial access sheath (5 French, F) for arterial blood pressure monitoring and one venous access sheath for interventional device implantation were used. Transseptal puncture was performed with a SL1 sheath and BRK1 Brockenbrough needle (St. Jude Medical, St. Paul, MN). Prior transseptal puncture heparin was administered to achieve an active clotting time of at least 250 s. A stiff guide wire (Cook Medical, Bloomington, IN) was placed in the left upper pulmonary vein (LUPV) and the transseptal sheath was removed and replaced by the Watchman™ (14 F), respectively ACP 2 (12 or 14 F) delivery sheath. Device allocation algorithm was performed according to latest consensus recommendations [21]. All patients underwent pre-procedural 2D/3D TEE to assess initially the anatomy, size and absence of thrombus or sludge on all recommended TEE views. Accordingly, peri-procedural imaging was based on fluoroscopy and 2D/3D TEE imaging in order to re-assess prior findings of the LAA and to decide which device size was appropriate in the individual patient. Fluoroscopy was performed using a 5 F pigtail catheter to visualize the LAA in at least 2 standard angulations (RAO 30°/10° cranial, RAO 30°/10° caudal). Regarding TEE, the following parameters of the LAA were assessed for appropriate sizing, as recommended for both devices [21]: LAA ostium, landing zone, angle of the LAA, depth of the LAA, differentiation of main lobes versus smaller side lobes. The device size was chosen at least 20 % larger than the measured diameters at the landing zone [22]. Post-implantation the compression of the devices was measured by 2D TEE and a device compression of at least 10 % was defined as valuable, next to lacking of relevant peri-device leaks (>5 mm), and no compression or attachment of neighboring structures, such as the circumflex coronary artery, mitral annulus or pulmonary veins. A tug test was performed repetitively before final liberation of the device as recommended. Access site was closed with an Angio-Seal™ Evolution™ (St. Jude Medical, St. Paul, MN) for the arterial access and with two ProGlide™ (Abbott Vascular, Santa Clara, CA) for the venous access and followed by a pressure band for 6 hours.

### Post-procedural measures

In accordance with our institutional protocol, acetylsalicylic acid (ASA) 100 mg/d was administered lifelong, clopidogrel for at least 6 months commencing the day of implantation with a loading dose of 250 mg, respectively 600 mg, if not taken before. The day after the procedure, a transthoracic echocardiogram (TTE) and a chest X-ray were performed to rule out device dislodgement and pericardial effusion, an ECG was performed to rule out a new bundle branch block (BBB) or atrioventricular (AV) block and repetitive thorough clinical examinations were carried out to rule out other clinical disorders.

### Outcome measures

The primary efficacy outcome measure of our in-hospital register was defined as technical success with a successful device implantation without relevant peri-device leaks (i.e., < 5 mm). Primary safety outcome measure was defined as the occurrence of bleeding events classified according to the BARC definition [23], pericardial effusion, device embolization, peri-procedural stroke and peri-procedural death. Events resulting in death, aggravated morbidity or prolonged hospitalization being associated with the procedure were defined as complications. Any events not being associated with the procedure were termed adverse events.

### Statistics

Statistical analyses were performed with SPSS Statistics (IBM, Armonk, NY). Descriptive statistics are given as medians (25^th^ and 75^th^ percentiles) or as total numbers with group-related percentages. Normal distribution of data was tested with the Kolmogorov-Smirnov test. In case of normal distribution, the *t*-test was applied to compare scaled data. Scaled variables not normally distributed were compared using the Mann-Whitney *U* test. Categorical variables were compared using the chi-squared test. Level of significance was set at *p* < 0.05 (two-tailed).

## Results

### Baseline characteristics

Baseline demographic and clinical characteristics of the study population including indications for implantation of LAA occlusion devices and risk-stratification according to CHA_2_DS_2_-VASc and HAS-BLED scores are shown in Table 1. Of 37 patients, 22 patients received the Watchman™ and 15 patients received the ACP 2 device. The most common indication was a prior history of bleedings (78.4 %) under treatment with OAC. 51.4 % of all patients were not treated with OAC, whereas patients with OAC were mostly treated with DOAC (66.7 %). Table 2 shows two-dimensional (2D) and functional echocardiographic measurements. As assessed by angiography, the majority of patients revealed a chicken-wing shaped LAA (67.6 %).

**Table 1:** Baseline characteristics of the study collective

	Watchman	ACP 2	All	*p* Value
Patients, n (%)	22 (59.5)	15 (40.5)	37 (100)	-
Male, n (%)	16 (72.7)	9 (60)	25 (67.6)	0.417
Age, y (IQR)	77 (70.75–81)	80 (76–83)	79 (71.5–81.5)	0.213
Height, cm (IQR)	170 (167.75–177)	170 (165–172)	170 (167–175)	0.090
Weight, kg (IQR)	80.5 (67.5–90)	83.5 (69.75–93.25)	81.5 (69.25–90)	0.531
Reason for not taking OAC, n (%)				
- GI bleeding	8 (36.4)	8 (53.8)	16 (43.2)	0.306
- ICB	4 (18.2)	2 (13.3)	6 (16.2)	0.694
- Muscle bleeding	2 (9.1)	0 (0)	2 (5.4)	0.230
- Other bleeding localization	2 (9.1)	3 (20)	5 (13.5)	0.341
- Refusal	3 (13.6)	0 (0)	3 (8.1)	0.136
- Other causes	3 (13.6)	2 (13.3)	5 (13.5)	1.000
AF type, n (%)				
- Paroxysmal	12 (54.5)	7 (46.7)	19 (51.4)	0.638
- Persistent	2 (9.1)	3 (20)	5 (13.5)	0.341
- Permanent	8 (36.4)	5 (33.3)	13 (35.1)	0.850
Heart rhythm at hospitalization, n (%)				
- SR	6 (27.3)	6 (40)	12 (32.4)	0.225
- AF	12 (54.5)	9 (60)	21 (56.8)	0.742
- PM	2 (9.1)	0 (0)	2 (5.4)	0.230
- unsp.	2 (9.1)	0 (0)	2 (5.4)	0.230
Prior PVI, n (%)	2 (9.1)	1 (6.7)	3 (8.1)	0.791
Hypertension, n (%)	21 (95.5)	15 (100)	36 (97.3)	0.403
Diabetes mellitus, n (%)	6 (27.3)	6 (40)	12 (32.4)	0.417
TIA, n (%)	4 (18.2)	0 (0)	4 (10.8)	0.080
Stroke, n (%)	1 (4.5)	2 (13.3)	3 (8.11)	0.336
Coronary artery disease, n (%)	13 (59.1)	8 (53.3)	21 (56.8)	0.729
Peripheral vascular disease, n (%)	3 (13.6)	1 (6.7)	4 (10.8)	0.503
Renal failure, n (%)	8 (36.4)	5 (33.3)	13 (35.1)	0.850
- GFR (IQR)	70 (43.5–70)	70 (41–70)	70 (42–70)	0.636
Liver failure, n (%)	3 (13.6)	1 (6.7)	4 (10.8)	0.503
Prior bleeding, n (%)	16 (72.7)	13 (86.7)	29 (78.4)	0.312
Labile INR, n (%)	2 (9.1)	1 (6.7)	3 (8.1)	0.791
CHA_2_DS_2_-VASc score (IQR)	4 (3–5)	5 (3–5)	5 (3–5)	0.292
HAS-BLED score (IQR)	4 (3–5)	4 (3–4)	4 (3–5)	0.538
Baseline OAC, n (%)				
- None	10 (45.5)	9 (60)	19 (51.4)	0.385
- Warfarin	3 (13.6)	3 (20)	6 (16.2)	0.606
- Rivaroxaban	6 (27.3)	2 (13.3)	8 (21.6)	0.312
- Dabigatran	1 (4.5)	1 (6.7)	2 (5.4)	0.779
- Apixaban	2 (9.1)	0 (0)	2 (5.4)	0.230
Cardiac device, n (%)				
- PM	4 (18.2)	3 (20)	7 (18.9)	0.890
- ICD	1 (4.5)	2 (13.3)	3 (8.1)	0.336

Values are given as medians (25^th^ and 75^th^ percentiles) or total numbers (percentage). *AF* atrial fibrillation, *GFR* glomerular filtration rate, *GI* gastrointestinal, *ICB* intracerebral bleeding, *ICD* implantable cardioverter defibrillator, *INR* international normalized ratio, *OAC* oral anticoagulation, *PM* pacemaker, *PVI* pulmonary vein isolation, *SR* sinus rhythm, *TIA* transient ischemic attack, *unsp.* unspecified

**Table 2:** Baseline echocardiographic data

	Watchman	ACP 2	All	*p* Value
LV function, n (%)				
- Normal	14 (63.6)^a^	14 (93.3)^a^	28 (75.5)	0.039
- Mild impairment	6 (27.3)^a^	0 (0)^a^	6 (16.2)	0.027
- Moderate impairment	1 (4.5)	0 (0)	1 (2.7)	0.403
- Severe impairment	1 (4.5)	1 (6.7)	2 (5.4)	0.779
LVEDD, mm (IQR)	49 (44–54.25)	45.5 (40–49.5)	46.5 (42.25–53.75)	0.096
Aortic valve, n (%)				
- Stenosis	2 (9.1)	2 (13.3)	4 (10.8)	0.638
- Regurgitation	10 (45.5)	3 (20)	13 (35.1)	0.111
Pulmonary valve, n (%)				
- Stenosis	0 (0)	0 (0)	0 (0)	-
- Regurgitation	0 (0)	1 (6.7)	1 (2.7)	0.220
Mitral valve, n (%)				
- Stenosis	2 (9.1)	1 (6.7)	3 (8.1)	0.791
- Regurgitation	14 (63.6)	11 (73.3)	25 (67.6)	0.536
Tricuspid valve, n (%)				
- Stenosis	0 (0)	0 (0)	0 (0)	-
- Regurgitation	12 (54.5)	10 (66.7)	22 (59.5)	0.461
Aortic bulb diameter, mm (IQR)	31 (29–35.25)	31 (27.75–35.25)	31 (29–35)	0.737
LAA (IQR)				
- Diameter, mm	48.5 (44.25–55)	40 (40–47.5)	46 (42.5–53)	0.193
- Plane, cm^2^	23 (18.5–28.25)	23 (14.5–28.75)	23 (17–28.5)	0.802
- Volume, ml	83.5 (66–101,75)	58.5 (46.75–99.5)	74.5 (57.5–101.75)	0.329
- Depth, mm	34 (27–38.75)	35 (27.5–40.75)	35 (28–40)	0.262
LAA ostial diameter, mm (IQR)				
- 0°	18 (16–23)	20 (13.5–22)	18 (16–22)	0.797
- 45°	18 (16.25–21.75)	19 (14–21)	18 (16–21)	0.727
- 90°	18 (16.75–20.25)	20 (14.75–21)	18.5 (16–21)	0.754
- 135°	20 (16.5–22.5)	21 (17.25–22)	20 (17.25–22)	0.569
Landing zone, mm (IQR)	17 (13.5–21.5)	16 (13.5–19.5)	16 (13.75–20.5)	0.556

Values are given as medians (25^th^ and 75^th^ percentiles) or total numbers (percentage). *LAA* left atrial appendage, *LV* left ventricular, *LVEDD* left ventricular end-diastolic dimension. ^a^Indicating significant difference

### Procedural data

Procedural data related to the implantation of LAA occlusion devices are summarized in Table 3. The primary efficacy outcome measure in the Watchman™ group was 90.9 %, whereas it was 93.3 % in the ACP 2 group (*p* = 0.791). Only the Watchman™ device device could not be implanted in two patients (9.1 %) either due to an anatomical mismatch or due to an incomplete occlusion of the LAA landing zone. In contrast, the ACP 2 device was implanted in all patients with one patient with incomplete ostial occlusion (6.7 %). More than one transseptal puncture was needed only in only one patient (2.7 %) due to a transseptal retrieval of an intra-atrial embolized Watchman™ occluder during the procedure. Pericardial effusion and subsequent intermittent circulatory failure with rapid hemodynamic stabilization without the need for cardiopulmonary resuscitation occurred in one patient of the Watchman™ group (2.7 %). All these results showed no statistically significant difference between the Watchman™ and the ACP 2 group.

**Table 3:** Procedural data

	Watchman	ACP 2	All	*p* Value
Success rate, n (%)	21 (95.5)	15 (100)	36 (97.3)	0.403
Complete ostial occlusion (i.e., gap < 5 mm), n (%)	20 (90.9)	14 (93.3)	34 (91.9)	0.791
Positioning attempts, n (IQR)	2.5 (1.75–3)	2 (1.5–5)	2 (2–3)	0.555
Changes of device size, n (IQR)	1 (1–1)	1 (1–2)	1 (1–1)	0.169
Final device size, mm (IQR)	24 (21–27)	25 (22–30)	24 (22–27)	-
Duration, min. (IQR)	122.5 (75–136.25)	100 (80–110)	110 (76–135)	0.069
Fluoroscopy time, min. (IQR)	11.7 (7.4–16.8)	12.7 (9.5–17.7)	12.4 (8.5–16.9)	0.244
Reference dose, Gy*cm2 (IQR)	80.5 (45.75–103.25)	62 (52–89)	64 (49–96)	0.842
Amount of contrast agent, ml (IQR)	165 (130–202.25)	140 (110–180)	157 (110–195)	0.833
LAA configuration, n (%)				
- Chicken-wing	14 (63.6)	11 (73.3)	25 (67.6)	0.536
- Windsock	3 (13.6)	1 (6.7)	4 (10.8)	0.503
- Broccoli	4 (18.2)	3 (20)	7 (18.9)	0.890
- Tub	1 (4.5)	0 (0)	1 (2.7)	0.403
Number of lobi, n (%)				
- 1	4 (18.2)	3 (20)	7 (18.9)	0.890
- 2	13 (59.1)	10 (66.7)	23 (62.2)	0.641
- 3	1 (4.5)	0 (0)	1 (2.7)	0.403
- Multi	4 (18.2)	2 (13.3)	6 (16.2)	0.694
Days on ICU/IMC, n (IQR)	1 (1–1)	1 (1–1)	1 (1–1)	0.327
Post-procedural days in hospital, n (IQR)	3 (2.25–4)	4 (2.75–5.25)	4 (2.75–5)	0.341

Values are given as medians (25^th^ and 75^th^ percentiles) or total numbers (percentage). *ICU* intensive care unit, *IMC* intermediate care unit

### In-hospital outcome and peri-procedural safety events

Table 4 shows all relevant data related to in-hospital outcome and peri-procedural safety events. None of the patients died due to the implantation of LAA occlusion devices. However, one patient with a preexisting highly reduced left ventricular function (ejection fraction 28 %) died from an acute heart failure linked to a severe urosepis 20 days after the implantation (i.e., adverse event). In another patient with non-ST elevation myocardial infarction (NSTEMI) and percutaneous coronary intervention (PCI) with stent implantation known clopidogrel non-response, DAPT with ASA and ticagrelor instead of clopidogrel was continued as an individual treatment attempt.

**Table 4:** Peri-procedural complications and safety events

	Watchman	ACP 2	All	*p* Value
Overall complications, n (%)	11 (50)	9 (60.0)	20 (54.1)	0.549
Major complications, n (%)	3 (13.6)	0 (0)	3 (8.1)	0.136
Device embolization, n (%)	1 (4.5)	0 (0)	1 (2.7)	0.403
Circulatory failure, n (%)	2 (9.1)	0 (0)	2 (5.4)	0.230
- With CPR	1 (4.5)	0 (0)	1 (2.7)	0.403
- Without CPR	1 (4.5)	0 (0)	1 (2.7)	0.403
Bleeding complications, n (%)	8 (36.4)	9 (60.0)	17 (48.6)	0.157
- Groin bruise	4 (18.2)	5 (33.3)	9 (24.3)	0.292
- Groin bleeding	3 (13.6)	3 (20.0)	6 (16.2)	0.606
- Pericardial effusion	1 (4.5)	0 (0)	1 (2.7)	0.403
- Other (dental)	0 (0)	1 (6.7)	1 (2.7)	0.220
Access-site bleedings, n (%)	7 (31.9)	8 (53.3)	15 (40.5)	0.191
- Venous access	0 (0.0)	1 (6.7)	1 (2.7)	0.220
- Arterial access	4 (18.2)	2 (13.3)	6 (16.2)	0.694
- Both access-sites	3 (13.6)	5 (33.3)	8 (21.6)	0.153
BARC score, each n (%)				
- Type 1	7 (31.8)	6 (40.0)	13 (35.1)	0.609
- Type 2	1 (4.5)	2 (13.3)	3 (8.1)	0.336
- Type 3a	0 (0.0)	1 (6.7)	1 (2.7)	0.220
- Type 3b	0 (0.0)	0 (0.0)	0 (0.0)	-
- Type 3c	0 (0.0)	0 (0.0)	0 (0.0)	-
- Type 4	-	-	-	-
- Type 5a	0 (0.0)	0 (0.0)	0 (0.0)	-
- Type 5b	0 (0.0)	0 (0.0)	0 (0.0)	-

Values are given as total numbers (percentage). *BARC score* Bleeding Academic Research Consortium score, *CPR* cardiopulmonary resuscitation

Severe conduction blocks, such as BBB or AV blocks, did not occur. Access site complications, such as groin bruise and bleedings at the access sites represented 75.0 % of all complications. Most of them were mild, corresponding to a BARC type 1 bleeding (76.5 % of all bleeding complications) and only 1 case (2.7 % of all patients) needed transfusion without the need for vascular surgery. The patient with a pericardial effusion (3 mm) in the Watchman™ group could be treated conservatively. Notably, neither peri-procedural nor in-hospital transient ischemic attack, stroke or device thrombosis occurred. Patients could have been discharged with a median of 4 days after the intervention. None of the surviving patients developed persistent neurological or heart-failure related disability.

## Discussion

AF is one of the main causes leading to ischemic stroke and persistent neurological disability. In almost 25 % of patients developing an ischemic stroke AF can be documented by ECG recording [3]. Therefore effective prevention of cerebral embolization represents the most important aspect for an optimal treatment of patients with AF. Besides the established vitamin K antagonists such as warfarin, innovative DOACs emerged as reliable treatment alternatives. In a current meta-analysis these drugs were shown to reduce significantly the risk of major cerebral bleedings and hemorrhagic stroke when compared to warfarin [7, 24]. Specific subsets of patients of patients are prone to develop severe bleedings as a consequence of OAC, for instance patients with malignoma, major gastrointestinal bleedings and geriatric patients with an increasing risk to fall in everyday life. Accordingly, most of the patients within the presented study revealed a history of severe bleedings leading to the decision to implant an LAA occlusion device. These patients revealed an increased median HAS-BLED score of 4 points and still revealed an increasing risk of developing stroke, as indicated by an increased median CHA_2_DS_2_-VASc score of 5 points corresponding to an estimated annual risk of thromboembolic stroke of about 6.7 % [4].

Relative contraindications against the use of OAC have been reported in about 20 % of patients with AF [25, 26]. Even patients without any contraindication against OAC are often not treated by the optimal antithrombotic drug regimes [27, 28]. Within the present study, half of the study patients were not anticoagulated previously due to relevant contraindications. In these patients exclusion of the LAA has become the therapy of choice in order to prevent fatal thromboembolic events apart from OAC [9, 29, 30]. Since we only enclosed patients with a high bleeding risk (a median HAS-BLED score of 4 points indicates “high risk” [6]) or refusal for OAC and, therefore, relative or absolute contraindication for OAC, DAPT with ASA and clopidogrel following implantation procedure was thought to be appropriate for these patients. The duration of DAPT lasted 6 months as previously been shown to be an effective and safe antithrombotic treatment [10].

Success rates of 95.5–100 %, respectively, were comparable to prior studies using the Watchman™ device [9, 10] or were even higher for the ACP 2 [12, 14]. Since complete coverage of the LAA ostium is crucial for preventing from embolization from the LAA, this was defined as a primary efficacy outcome measure, which was also beyond 90 % for both devices.

The documented high efficacy appears to be associated with a careful and appropriate selection of device sizes according to the pre- and peri-procedural measurements of LAA dimensions being assessed by a multi-modal imaging approach with angiography and TEE imaging [14, 22] allowed a safe and accurate peri-procedural guiding for an optimal positioning and adaption. Diameters of the LAA ostium, landing zone, angulation, deepness and volume appeared most similar in recommended angulations (0, 45, 90 and 135°) with a tendency for larger orifice diameters being measured at 135° of angulation [31]. The median size of the devices was about 40 % larger than the median landing as assessed by TEE, surpassing the 20 % range of recommended oversizing [22].

One patient revealed a flat tub-shaped LAA morphology, which made implantation of both devices impossible, whereas all other typical LAA morphologies were able to be accessed by LAA occlusion devices. Even the relatively frequent occurrence of the chicken-wing morphology, described as often being a challenging morphology for transcatheter occlusion device implantation [32], was not seen to decrease the high success rates of both devices. Time of fluoroscopy was comparable to prior studies [18, 19] and did not differ significantly between both groups despite the fact that decision making algorithm in our study regarding accurate measurements of the LAA and selection of the adequate device size was based on peri-procedural 2D TEE in combination with angiography. 3D TEE has been described as an additional imaging technique for optimal visualization of cardiac anatomy during percutaneous cardiac interventions [22, 33]. However, efficacy data evaluating TEE (either 2D or 3D) versus sole or combined angiography during implantation of LAA occlusion devices are not available at present. Since the procedures were performed by experienced interventional cardiologists, well practiced in LAA closure device implantation, we could not find a certain learning curve neither concerning procedural time nor related to peri-procedural complications [34].

Major peri-procedural complications were rare (8.1 %) and did not reveal persistent disability. At 40.5 %, groin hematomas and groin bleedings occurred more frequently compared to recently published data [10, 18, 20]. However, the majority of access site bleedings were minor hematomas (80.0 % of all access-site complications corresponded to a BARC type 1 bleeding). The majority of patients suffering from access-site complications was affected by combined arterial and venous access-site bleedings (21.6 % of all patients). As must be expected, the arterial access-site was the second most common bleeding site (16.2 % of all patients). Since an arterial access during LAA closure device implantation procedure is not mandatory [35], a certain number of access-site bleedings may be avoidable if this access way would be abandoned. Notably, the arterial access allows a safer performance [35] at the expense of some additional mild hematomas. Only one patient was in need of blood transfusion, whereas vascular surgery was never needed. With respect to major complications, one case of circulatory failure was linked to a post-procedural detected pericardial effusion (3 mm), which could be treated conservatively. Early device thrombosis followed by TIA or stroke did not occur in our population suggesting that DAPT initiated by a loading dose might be effective to prevent thromboembolism at the intraluminal device site prior to neo-endothelialization. One patient with intra-procedural device dislodgement in the left atrium (Watchman™ group) which could be successfully retrieved by a veno-venous double lasso interventional technique.

### Limitations of the study

This study is based on observational registry data concerning a relative small real-life patient population and was not intended to reliable calculate significant differences between both device types. Comparison results of both devices are for informative reasons only. The aim was to demonstrate essential peri-interventional efficacy and safety data concerning LAA occlusion device implantation in general. A subsequent enlarged set of this registry data might be able to evaluate further differences between both devices.

As this observational study focused on the peri-procedural time, the follow-up period was limited to the discharge from hospital.

The application of any of the two devices was not randomly assessed and was based on the operator’s discretion being mainly based on LAA anatomic considerations. In contrast, this more individualized and clinically driven decision algorithm might have strongly influenced the high achievement of primary efficacy outcome measure, which realistically reflects clinical and interventional practice in experienced centers.

## Conclusions

Transcatheter implantation of LAA occlusion devices appears to be a feasible and safe percutaneous cardiac intervention with a rare occurrence of major complications and a high success rate in a real-life patient population.

### Ethics, consent and permissions

Written informed consent was obtained from all study patients. The study was carried out according to the principles of the declaration of Helsinki and was approved by the medical ethics committee II of the Faculty of Medicine Mannheim, University of Heidelberg, Germany.

### Availability of supporting data

Sharing of additional data is not necessary, because all relevant patient data are presented in this article.
